# Associations between serum total bilirubin, obesity and type 2 diabetes

**DOI:** 10.1186/s13098-021-00762-0

**Published:** 2021-12-07

**Authors:** Yuan Wei, Chaoqun Liu, Fangfang Lai, Shan Dong, Haiyan Chen, Li Chen, Liping Shi, Fengfeng Zhu, Chuangbiao Zhang, Xiuxiu Lv, Shuang Peng, Guang Hao

**Affiliations:** 1 Center for Scientific Research and Institute of Exercise and Health, Guangzhou Sport University, Guangzhou, 510500 China; 2 Department of Nutrition, School of Medicine, Jinan University, Guangzhou, 510632 China; 3 Department of Pediatrics, Renmin Hospital of Wuhan University, 238 Jiefang Road, Wuhan, 430060 China; 4 Guangzhou First People’s Hospital, The Second Affiliated Hospital of South China University of Technology, Guangzhou, 510180 China; 5 Department of Endemic Disease, Guangzhou Center for Disease Control and Prevention, Guangzhou, 510632 China; 6 Georgia Prevention Institute, Department of Medicine, Medical College of Georgia, Augusta University, Augusta, GA 30912 USA; 7 Department of Urology, The First Affiliated Hospital of Jinan University, Guangzhou, 510630 China; 8 Department of Hepatobiliary and Pancreas Surgery, The First Affiliated Hospital of University of South China, Hengyang, 421001 China; 9 Department of Endocrinology, The First Affiliated Hospital of Jinan University, Guangzhou, 510632 China; 10 Department of Pathophysiology, School of Medicine, Jinan University, 601 Huangpu Avenue West, Guangzhou, 510632 Guangdong China; 11 Department of Public Health and Preventive Medicine, School of Medicine, Jinan University, 601 West Huangpu Ave, Guangzhou, 510632 Guangdong China

**Keywords:** Bilirubin, Obesity, Diabetes, Glycohemoglobin, Insulin resistance

## Abstract

**Background:**

This study aims to examine the cross-sectional association between serum total bilirubin (STB) and type 2 diabetes (T2D) risk in the general population, and whether obesity could moderate this association.

**Methods:**

We used data from the 1999–2018 National Health and Nutrition Examination Surveys (NHANES), including a total of 38,641 US adult participants who were 18 years or older. The STB was classified as the low, moderate, and high groups according to tertiles.

**Results:**

We found that participants with lower STB had a significantly higher risk of T2D than those with moderate (OR = 0.81; 95% CI 0.74, 0.89; *P* < 0.001) and high (OR = 0.65; 95% CI 0.59, 0.73; *P* < 0.001) STB. Also, a significant interaction between body mass index (BMI) and STB on T2D was observed (*P* < 0.001). Stratified analysis showed that low STB was associated with a 20% and 27% decrease of T2D risk for moderate and high STB groups in obese patients, however, these effect estimates were smaller in the population with lower BMI (< 30 kg/m^2^). Similar associations of STB with glycohemoglobin and insulin resistance were observed.

**Conclusion:**

This study suggests that STB is associated with an elevated risk of T2D. More importantly, we reported for the first time that BMI may moderate the association between bilirubin and T2D.

**Supplementary Information:**

The online version contains supplementary material available at 10.1186/s13098-021-00762-0.

## Introduction

Type 2 diabetes (T2D) is a chronic disease that has become a serious issue in morbidity and health expenditures [1, 2]. As a leading cause of death, global health expenditure relating to diabetes reached appropriately USD 727 billion in 2017 [3]. In 2019, it was estimated that 463 million people are living with diabetes worldwide and the number is projected to increase by 25% in 2030 and 51% in 2045, reflecting a continuously rising global burden [4].

The predisposition to develop T2D is believed to be influenced by multiple factors including genetic, environmental, and epigenetic modifications [5]. Risk factors for diabetes such as obesity, age, physical inactivity, dietary patterns have been well characterized and studied but the complex etiology of diabetes is still not fully understood [6]. In the context of the rapid rise of global costs of diabetic health care and management, it is therefore imperative to identify novel risk factors of diabetes, which may help in the screening of high-risk populations and the introduction of early intervention strategies to prevent escalation of this chronic disease.

Bilirubin, as an end-product of heme catabolism in the systemic circulation, has been shown to have antioxidant effects [3, 7] Most previous studies suggested that bilirubin was negatively associated with a high risk of diabetes and its chronic complications [7–12]. On the other hand, research has suggested that there may be a bidirectional association between STB and obesity [13, 14]. The aim of this study, therefore, was to evaluate the cross-sectional associations of serum total bilirubin (STB) with the risk of T2D and the interactions between STB and other risk factors using a large sample from the National Health and Nutrition Examination Survey (NHANES).

## Research design and methods

### Design and participants

The NHANES is conducted by the National Center for Health Statistics (NCHS) and the Centers for Disease Control and Prevention. It included a stratified multistage probability sample representative of the civilian non-institutionalized US population. Detailed descriptions of the survey design and data collection procedures are available elsewhere [15]. Since 1999, the demographic, socioeconomic, health-related data, including laboratory measurements of serum sodium, fasting blood glucose, and glycohemoglobin, etc., have been collected via an in-home interview and a visit to a mobile examination center. The research ethics boards of NCHS approved all protocols.

In the primary analysis, we used data from the 1999–2018 NHANES (including 10 cycles). The following selection criteria were used: (1) aged ≥ 18 years older; (2) data available on serum total bilirubin, fasting blood glucose, glycohemoglobin, total cholesterol (TC), age, sex, race, body mass index (BMI), smoking status, alcohol consumption, recreational physical activity (RPA), education attainment, and poverty income ratio. Also, potential Gilbert syndrome group (total bilirubin > 34.2 µmol/L, aspartate aminotransferase < 80 IU/L, alanine transaminase < 80 IU/L, gamma glutamyl transpeptidase < 80 IU/L, and no self-reported history of liver disease) and potential hepatobiliary disease group (total bilirubin > 34.2 µmol/L or aspartate aminotransferase > 80 IU/L or aspartate aminotransferase > 80 IU/L or serum albumin > 3.5 g/dL or positive self-reported history of liver disease) were excluded to avoid confounding from liver impairment [16]. Finally, a total of 38,641 US adult participants were eligible for analysis. In the secondary analysis, we also examined the associations of STB with and insulin resistance (estimated by homeostasis model assessment of insulin resistance, HOMA-IR).

### Measurements and definitions

Self-reported insulin uses and diabetes medication use was obtained by a medical history questionnaire administered by trained study staff. T2D was defined as glycohemoglobin ≥ 6.5%, fasting blood glucose ≥ 126 mg/dL, or self-reported use of insulin or other diabetes medication. Glucose concentration was determined by a hexokinase method, which is an endpoint enzymatic method with a sample blank correction. Because there were changes in laboratory methods and laboratory equipment for insulin and blood glucose across the different waves of tests, regression equations recommended by the NHANES Analytic Guidelines were used for an adjustment accordingly. Homeostasis model assessment of insulin resistance (HOMA-IR) was calculated with the formula: fasting blood glucose (mmol/L) times fasting serum insulin (mU/L) divided by 22.5 [17]. STB levels were measured using Hitachi and Beckman analyzers (range of values: 0.1–30.0 mg/dL). The STB was classified as the low, moderate, and high groups according to tertiles. TC was measured by an enzymatic assay. Serum low-density lipoprotein cholesterol (LDL-D) levels were derived on study participants who were examined in the morning session only, and it was calculated according to the Friedewald calculation [18]. BMI was calculated using weight in kilograms divided by height in meters squared. Abdominal obesity was defined as a waist circumference of at least 102 cm for males and at least 88 cm for females [19].

The poverty income ratio is the ratio of a family's income to the US Census Bureau's poverty threshold, which is adjusted for family size and is updated annually for inflation. The poverty income ratio was used as the indicator of socioeconomic status in the analyses. Participants who did vigorous/moderate RPA over the past 30 days (1999–2006 cycle)/ in a typical week (2007–2018 cycle) for at least 10 min that cause heavy sweating, or large increases in breathing or heart rate were defined as vigorous/moderate RPA, and others were defined as light RPA [20]. Participants were categorized as never smokers (individuals who have smoked < 100 cigarettes in life), former smokers (having smoked > 100 cigarettes in life but do not currently smoke), and current smokers. Significant alcohol use was defined as > 30 g/day for men and > 20 g/day for women [21]. The presence of active liver disease was determined by the subject’s answer to the questions ‘Has a doctor or other health professional ever told you that you have liver disease?’ and ‘Do you still have a liver condition?’.

### Statistical analysis

Appropriate sample weights were used to account for oversampling and nonresponse to provide nationally representative results, as recommended by NHANES Analytic Guidelines. Continuous variables were presented as mean and 95% confident interval (CI), whereas categorical variables were presented as percentage (%) and 95%CI. Chi-squared tests or ANOVA were used to compare the characteristics among three STB groups. Logistic regression was used to calculate the odds ratio (OR) and 95% confidence interval (CI) for the associations of STB and T2D. Linear regression was used to calculate the β and 95% CI for the associations of STB with fasting blood glucose, glycohemoglobin, and insulin resistance (participants with diabetes medications were excluded). Univariate analyses were performed in Model 1; age, sex, and race were adjusted in Model 2; BMI, TC, RPA, marital status, education attainment, and poverty income ratio were further adjusted in Model 3. A restricted cubic regression spline was used to test the nonlinear associations of STB with T2D and its biomarkers. In a sensitivity analysis, 2,849 participants with coronary heart disease and stroke were excluded to avoid confounding from the effects of secondary prevention. We presented the stratified results by survey period in another sensitivity analysis. Also, LDL-C and lipid lowering medicine were adjusted to test the robustness of the association (16.280 participants were available for this analysis). The interaction between serum STB and BMI, as well as their interactions with age and sex, were also tested. Furthermore, the mediation effect of c-reactive protein on the association between STB and T2D was examined (gsem package). All data analyses were performed using Stata software version 12.1 (STATA Corp., TX, US). A two-sided *P* < 0.05 was considered statistically significant.

## Results

A total of 38,641 participants were eligible for our primary analysis. Overall, their mean age was 46.5 years, and 52.4% were female. Table 1 showed the percentage of diabetics/participants within each of the STB groups in the present study. There were significant differences among low, moderate, and high STB groups except for age (*P* > 0.05).

**Table 1:** Characteristics of the study

Variable	Low (< 8.6 µmol/L) (n = 13,481)	Moderate (8.6–12.0 µmol/L) (n = 12,714)	High (≥ 12 µmol/L) (n = 12,446)	*P* value
Age (years)	46.1 (45.5–46.6)	46.9 (46.5–47.4)	46.4 (45.9–46.9)	0.373
Sex (Females, %)	68.0 (66.9–69.1)	54.4 (53.3–55.5)	34.6 (33.6–35.6)	< 0.001
Race (%)				
Non-Hispanic white	65.1 (62.4–67.7)	70.4 (68.1–72.6)	74.6 (72.6–76.5)	< 0.001
Non-Hispanic black	12.8 (11.2–14.6)	10.4 (9.3–11.7)	7.7 (6.8–8.6)	
Mexican–American	8.5 (7.3–9.9)	7.8 (6.7–9.0)	6.8 (5.8–7.9)	
Other	13.6 (12.3–15.0)	11.4 (10.3–12.5)	10.9 (9.8–12.2)	
Body mass index (kg/m^2^)	29.8 (29.6–30.0)	28.5 (28.3–28.7)	27.5 (27.3–27.7)	< 0.001
Smoking (%)				
Never	53.2 (51.9–54.6)	53.2 (51.7–54.7)	57.2 (55.8–58.6)	< 0.001
Former	20.3 (19.3–21.4)	22.5 (21.5–23.6)	24.0 (22.9–25.1)	
Current	26.5 (25.0–28.0)	24.2 (22.9–25.5)	18.8 (17.7–20.0)	
Significant alcohol use (%)	27.8 (26.7–28.9)	33.0 (31.7–34.2)	32.1 (30.8–33.4)	< 0.001
Marital status (%)	53.2 (51.6–54.7)	57.1 (55.6–58.6)	61.7 (60.2–63.2)	< 0.001
Education attainment				
< 9 years	5.5 (5.0–6.2)	5.6 (5.1–6.1)	4.8 (4.3–5.3)	< 0.001
9–11 years	11.1 (10.4–11.9)	11.6 (10.7–12.5)	8.9 (8.1–9.7)	
12 years	25.1 (23.9–26.3)	23.9 (22.9–25.0)	21.8 (20.5–23.1)	
> 12 years	58.2 (56.5–59.9)	58.9 (57.3–60.5)	64.6 (62.8–66.3)	
Vigorous/Moderate RPA (%)	53.9 (52.3–55.4)	59.8 (58.2–61.4)	65.6 (63.9–67.3)	< 0.001
Poverty income ratio	2.8 (2.8–2.9)	3.0 (3.0–3.1)	3.3 (3.2–3.3)	< 0.001
TC (mmol/L)	5.0 (5.0–5.0)	5.1 (5.1–5.1)	5.1 (5.1–5.1)	< 0.001

*PRA* recreational physical activity, *TC* total cholesterol

The STB was classified as the low, moderate, and high groups according to tertiles

**Fig. 1:**
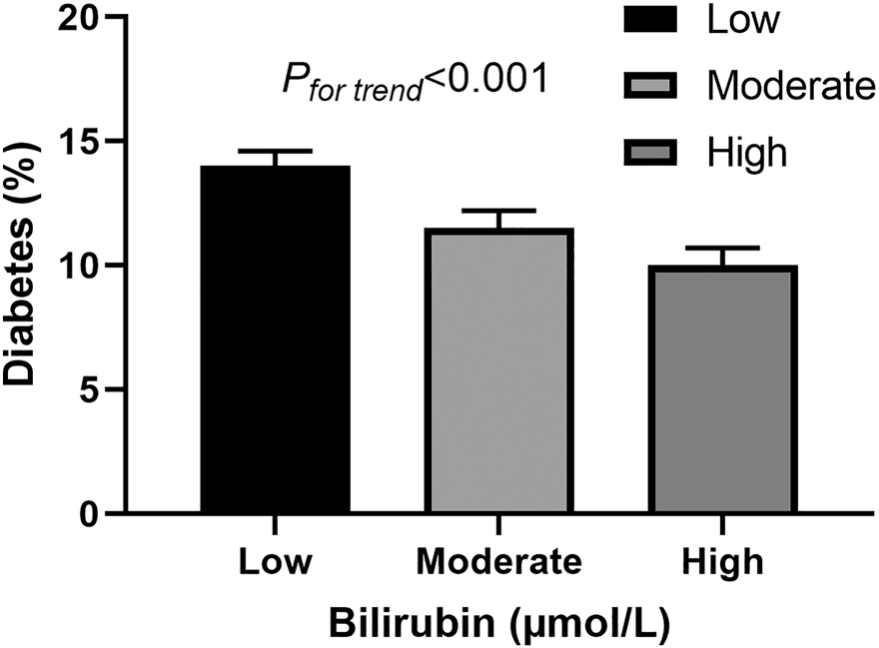
The proportions of diabetes by serum total bilirubin tertiles

The prevalence of T2D in the low STB group was significantly higher (*P* < 0.001): 13.2%, 11.0%, and 9.0% in low, moderate, and high STB groups (Fig. 1). Participants with lower STB had a significantly higher risk of T2D than those with moderate (OR = 0.81; 95% CI 0.74, 0.89; *P* < 0.001) and high (OR = 0.65; 95% CI 0.59, 0.73; *P* < 0.001) STB. When further adjusted for age, sex, race, smoking, significant alcohol use, BMI, TC, RPA, marital status, education attainment, and poverty income ratio, the results were similar: OR = 0.87; 95% CI 0.79, 0.85; *P* = 0.004 for moderate STB group, and OR = 0.74; 95% CI 0.65, 0.84; *P* < 0.001 for high STB group (Table 2). In a sensitivity analysis, after excluding 2,849 participants with coronary heart disease and stroke, the results were unchanged (Additional file 1: Table S1). Also, the associations in different time periods remained similar (Additional file 1: Table S2). In another sensitivity analysis, after adjusting LDL-C and lipid lowering medicine, the results remained similar (Additional file 1: Table S3). There was no evidence of non-linearity (*P* > 0.001), and low STB was linearly associated with a higher risk of T2D (*P* for trend < 0.001).

**Table 2:** The associations between serum total bilirubin and type 2 diabetes

Serum total bilirubin	OR	95%CI	*P* value
Model 1			
Low	Reference		
Moderate	0.81	0.74, 0.89	< 0.001
High	0.65	0.59, 0.73	< 0.001
*P* for trend	< 0.001		
Model 2			
Low	Reference		
Moderate	0.75	0.69, 0.83	< 0.001
High	0.58	0.52, 0.65	< 0.001
*P* for trend	< 0.001		
Model 3			
Low	Reference		
Moderate	0.87	0.79, 0.85	0.004
High	0.74	0.65, 0.84	< 0.001
*P* for trend	< 0.001		

*OR* Odds ratio, *CI* confidence intervals

Model 1 unadjusted

Model 2 adjusted for age, sex, and race

Model 3 adjusted for age, sex, race, smoking, significant alcohol use, body mass index, total cholesterol, recreational physical activity, marital status, education attainment, and poverty income ratio

There was an interaction between BMI with STB (*P* < 0.001) for the risk of T2D, which suggests that BMI may moderate the associations between bilirubin and T2D. In BMI-stratified analysis, we found that low STB was associated with a 20% and 27% decrease of T2D risk for moderate and high STB groups in obese patients (BMI ≥ 30), however, these effect estimates were smaller in the population with lower BMI (BMI < 30) (Table 3). We further conducted the stratified analysis by BMI and waist circumference, overall, low STB was associated with a decrease of T2D risk in different strata, although the associations of STD with T2D in population with normal/over-weight (BMI < 30) central obesity did not reach statistical significance (Additional file 1: Table S4).

**Table 3:** The associations between serum total bilirubin and type 2 diabetes by body mass index

Serum total bilirubin	Prevalence (%)	OR	95%CI	*P* value
BMI < 25 (n = 11,721)				
Low	5.2	Reference		
Moderate	4.6	0.92	0.73, 1.17	0.496
High	3.7	0.72	0.57, 0.92	0.009
*P* for trend		0.008		
BMI:25–30 (n = 13,188)				
Low	10.1	Reference		
Moderate	9.6	0.92	0.77, 1.12	0.412
High	7.7	0.73	0.58, 0.91	0.009
*P* for trend		0.005		
BMI ≥ 30 (n = 13,732)				
Low	20.5	Reference		
Moderate	18.6	0.80	0.70, 0.92	0.001
High	17.8	0.73	0.61, 0.86	< 0.001
*P* for trend		< 0.001		

*OR* Odds ratio, *CI* confidence intervals

Adjusted for age, sex, race, smoking, significant alcohol use, body mass index, total cholesterol, recreational physical activity, marital status, education attainment, and poverty income ratio

For glycohemoglobin, we found that the participants with low STB had a higher level than those with moderate and high STB groups (5.64, 5.55, and 5.45 mmol/L, *P* < 0.001). After adjusting for other covariates, the results also showed that low STB was significantly associated with glycohemoglobin for moderate STB group (β = − 0.041; 95% CI − 0.059, − 0.024; *P* < 0.001) and high group (β = − 0.098; 95% CI − 0.115, − 0.080; *P* < 0.001). Likewise, a linear association between STB and glycohemoglobin was also observed (Additional file 1: Table S5). We also found a similar association between STB and insulin resistance (Additional file 1: Table S6). However, the association between STB and fasting blood glucose level did not reach statistical significance (*P* > 0.05) (Additional file 1: Table S5).

Mediation analysis showed that c-reactive protein mediated 17.4% (*P* = 0.018) and 3.4% (*P* = 0.030) of the associations of STB with blood glucose and glycohemoglobin (Additional file 1: Figure S1).

## Discussion

We found that lower STB was significantly associated with a higher risk of T2D. Also, we reported, for the first time, a significant interaction between BMI and STB in diabetics, suggesting that BMI may moderate the association between bilirubin and T2D.

Many cross-sectional studies have reported that STB was inversely associated with an increased risk of T2D. For example, Wu et al*. *[22] reported that total bilirubin concentrations were inversely associated with hyperinsulinemia, insulin resistance, and hyperglycemia in a middle-aged and elderly Chinese population. Several cohort studies also showed a negative association between total bilirubin and the risk of T2D. In a recent study, Kawamoto et al. [23] found that STB was significantly and inversely associated with incident metabolic syndrome in both the cross-sectional and prospective analysis.

Previous studies have also shown that STB increases insulin resistance and glycohemoglobin level/concentration [8, 9, 24]. However, the association of total bilirubin with fasting blood glucose is inconsistent [22, 25–27]. Similar to some of the studies [25, 26], we did not find a significant association between STB and fasting blood glucose, which also indicates that the negative association between total bilirubin and the risk of T2D and metabolic syndrome may due to anti-oxidative and anti-inflammatory properties of bilirubin. In line with these findings, we also find a minor association between STB and T2D in the population with lower BMI (BMI < 30) and/or lower waist circumference, in whom the inflammation and oxidative stress may be less than in obese patients. On the other hand, we reported that c-reactive protein mediated 17.4% and 3.4% of the associations of STB with blood glucose and glycohemoglobin.

Previous studies have demonstrated that bilirubin is independently and inversely associated with BMI or adiposity [28, 29]. We further found a significant interaction between BMI and STB on T2D, suggesting that STB and obesity may have a joint effect on T2D, but the mechanisms are unclear. Bilirubin could also improve the lipid profile (including our data, Additional file 1: Table S7), leptin, and increases adiponectin [30], which may explain the large effect of bilirubin on T2D in obese. Another explanation possibly is that the anti-oxidative and anti-inflammatory character make bilirubin a more obvious effect on inflammatory diseases, such as T2D and obesity [31]. The association between bilirubin and obesity may be bidirectional. Obesity may decrease STB level via altering gut microbiota [32], while bilirubin may also protect against insulin resistance by ameliorating visceral obesity and adipose tissue inflammation [14].

## Limitations and strengths

A major strength of our study is the large sample size and performed in the general population. Also, we reported for the first time an interaction between obesity and total bilirubin on the risk of T2D. There were also several limitations. One limitation is that the cross-sectional nature of this study precludes the inference of the cause-effect relationship. Another limitation is that this study did not distinguish between type 1 and type 2 diabetes. Although the previous studies also reported the potential beneficial effects of bilirubin in type 1 diabetes [33]. Considering the lower proportion of type 1 diabetes, our results may not extrapolate to the patients with type 1 diabetes. Also, we were not able to examine the effects of indirect fraction and the direct fraction on the risk of T2D due to the lack of fractionation data, so the different fractionations of bilirubin should be further studied.

## Conclusion

In conclusion, this study found that lower STB is associated with an elevated risk of T2D in the general population. We for the first time report that there is an interaction between obesity and total bilirubin on T2D, suggesting that BMI may moderate the association between bilirubin and T2D. Our study may provide a novel insight into the identification of populations at high risk of T2D. Further cohort studies are warranted to verify these associations and elucidate the role of adiposity in the association between bilirubin and T2D.

## Supplementary Information

**Additional file 1: Table S1.** The associations of serum total bilirubin with type 2 diabetes (sensitivity analysis 1). **Table S2.** The associations between serum total bilirubin and type 2 diabetes by body mass index (sensitivity analysis 2). **Table S3.** The associations of serum total bilirubin with type 2 diabetes (sensitivity analysis 3). **Table S4.** The associations between serum total bilirubin and type 2 diabetes by body mass index and waist circumference. **Table S5.** The associations of serum total bilirubin with glycohemoglobin and fasting blood glucose. **Table S6.** The associations of serum total bilirubin with HOMA-IR. **Table S7.** The associations of serum total bilirubin with lipid profile. **Figure S1.** The mediation model of c-reactive protein in the association of serum total bilirubin with glucose and glycohemoglobin. A: serum glucose; B: glycohemoglobin. In the mediation analysis, patients with self-reported use of insulin or other diabetes were excuded, finally, 14,449 paricipants with c-reactive protein data were used.

## Data Availability

The data of this study are available at https://www.cdc.gov/nchs/nhanes/index.htm.
