# Supplementary material for: Associations between serum total bilirubin, obesity and type 2 diabetes
Source: Diabetol Metab Syndr. 2021 Dec 7;13:143. doi: 10.1186/s13098-021-00762-0 (PMC8650363; doi:10.1186/s13098-021-00762-0)
Supplement: Supplementary file 1 — Additional file 1: Table S1. The associations of serum total bilirubin with type 2 diabetes (sensitivity analysis 1). Table S2. The associations between serum total bilirubin and type 2 diabetes by body mass index (sensitivity analysis 2). Table S3. The associations of serum total bilirubin with type 2 diabetes (sensitivity analysis 3). Table S4. The associations between serum total bilirubin and type 2 diabetes by body mass index and waist circumference. Table S5. The associations of serum total bilirubin with glycohemoglobin and fasting blood glucose. Table S6. The associations of serum total bilirubin with HOMA-IR. Table S7. The associations of serum total bilirubin with lipid profile. Figure S1. The mediation model of c-reactive protein in the association of serum total bilirubin with glucose and glycohemoglobin. A: serum glucose; B: glycohemoglobin. In the mediation analysis, patients with self-reported use of insulin or other diabetes were excuded, finally, 14,449 paricipants with c-reactive protein data were used. [file 13098_2021_762_MOESM1_ESM.docx]

| Table S1. The associations of serum total bilirubin with type 2 diabetes (sensitivity analysis 1) | | | |
| --- | --- | --- | --- |
| Serum total bilirubin | OR | 95%CI | *P* value |
| Low | Reference |  |  |
| Moderate | 0.87 | 0.79, 0.96 | 0.006 |
| High | 0.76 | 0.66, 0.87 | 0.005 |
| *P* for trend | <0.001 |  |  |
| CI=confidence intervals  Adjusted for age, sex, race, smoking, significant alcohol use, body mass index, total cholesterol, recreational physical activity, marital status, education attainment, and poverty income ratio.  2,849 participants with coronary heart disease and stroke were excluded. | | | |

| Table S2. The associations between serum total bilirubin and type 2 diabetes by body mass index (sensitivity analysis 2) | | | |
| --- | --- | --- | --- |
| Serum total bilirubin | OR | 95%CI | *P* value |
| **NHANES 1999-2008** |  |  |  |
| Low | Reference |  |  |
| Moderate | 0.82 | 0.68, 0.99 | 0.043 |
| High | 0.69 | 0.57, 0.85 | 0.001 |
| *P* for trend | <0.001 |  |  |
| **NHANES 2009-2018** |  |  |  |
| Low | Reference |  |  |
| Moderate | 0.92 | 0.83, 1.01 | 0.087 |
| High | 0.81 | 0.68, 0.96 | 0.019 |
| *P* for trend | 0.015 |  |  |
| OR=Odds ratio; CI=confidence intervals  Adjusted for age, sex, race, smoking, significant alcohol use, body mass index, total cholesterol, recreational physical activity, marital status, education attainment, and poverty income ratio. | | | |

| Table S3. The associations of serum total bilirubin with type 2 diabetes (sensitivity analysis 3) | | | |
| --- | --- | --- | --- |
| Serum total bilirubin | OR | 95%CI | *P* value |
| Low | Reference |  |  |
| Moderate | 0.85 | 0.72, 1.00 | 0.053 |
| High | 0.83 | 0.69, 1.00 | 0.053 |
| *P* for trend | <0.001 |  |  |
| 16.280 participants were available for low density lipoprotein cholesterol and lipid lowering medicine  CI=confidence intervals  Adjusted for age, sex, race, smoking, significant alcohol use, body mass index, low density lipoprotein cholesterol, recreational physical activity, marital status, education attainment, and poverty income ratio. | | | |

| Table S4. The associations between serum total bilirubin and type 2 diabetes by body mass index and waist circumference | | | |
| --- | --- | --- | --- |
| Serum total bilirubin | OR | 95%CI | *P* value |
| **BMI<25** |  |  |  |
| **Without abdominal obesity (n=** **10,416)** |  |  |  |
| Low | Reference |  |  |
| Moderate | 0.86 | 0.65, 1.12 | 0.260 |
| High | 0.66 | 0.50, 0.88 | 0.005 |
| *P* for trend | 0.005 |  |  |
| **Abdominal obesity (n=1,008)** |  |  |  |
| Low | Reference |  |  |
| Moderate | 1.17 | 0.65, 2.08 | 0.602 |
| High | 0.92 | 0.49, 1.73 | 0.791 |
| *P* for trend | 0.844 |  |  |
| **BMI:25-30** |  |  |  |
| **Without abdominal obesity (n=** **5,961)** |  |  |  |
| Low | Reference |  |  |
| Moderate | 0.68 | 0.48, 0.96 | 0.030 |
| High | 0.55 | 0.39, 0.77 | 0.001 |
| *P* for trend | 0.001 |  |  |
| **Abdominal obesity (n=6,897)** |  |  |  |
| Low | Reference |  |  |
| Moderate | 1.07 | 0.86, 1.32 | 0.551 |
| High | 0.85 | 0.66, 1.11 | 0.236 |
| *P* for trend | 0.241 |  |  |
| **BMI≥30** |  |  |  |
| **Without abdominal obesity (n=** **475)** |  |  |  |
| Low | Reference |  |  |
| Moderate | 1.20 | 0.38, 3.81 | 0.750 |
| High | 1.10 | 0.50, 2.41 | 0.802 |
| *P* for trend | 0.833 |  |  |
| **Abdominal obesity (n=12,802)** |  |  |  |
| Low | Reference |  |  |
| Moderate | 0.81 | 0.71, 0.93 | 0.003 |
| High | 0.73 | 0.61, 0.87 | <0.001 |
| *P* for trend | <0.001 |  |  |
| OR=Odds ratio; CI=confidence intervals; Abdominal obesity: men, waist circumference≥102 cm; women, waist circumference≥88 cm;  Adjusted for age, sex, race, smoking, significant alcohol use, total cholesterol, recreational physical activity, marital status, education attainment, and poverty income ratio.  1082 participants were excluded due to missing data on waist circumference. | | | |

| Table S5. The associations of serum total bilirubin with glycohemoglobin and fasting blood glucose | | | |
| --- | --- | --- | --- |
| Serum total bilirubin | β | 95%CI | *P* value |
| **Glycohemoglobin** |  |  |  |
| Low | Reference |  |  |
| Moderate | -0.041 | -0.059, -0.024 | <0.001 |
| High | -0.098 | -0.115, -0.080 | <0.001 |
| *P* for trend | <0.001 |  |  |
| **Fasting blood glucose** |  |  |  |
| Low | Reference |  |  |
| Moderate | -0.031 | -0.065, 0.003 | 0.076 |
| High | -0.023 | -0.059, 0.013 | 0.211 |
| *P* for trend | 0.211 |  |  |
| CI=confidence intervals  Adjusted for age, sex, race, smoking, significant alcohol use, body mass index, total cholesterol, recreational physical activity, marital status, education attainment, and poverty income ratio.  3,813 diabetic patients with diabetic medications were excluded. | | | |

| Table S6. The associations of serum total bilirubin with HOMA-IR | | | |
| --- | --- | --- | --- |
| Serum total bilirubin | β | 95%CI | *P* value |
| Low | Reference |  |  |
| Moderate | -0.974 | -1.784, -0.165 | 0.019 |
| High | -1.886 | -2.867, -0.905 | <0.001 |
| *P* for trend | <0.001 |  |  |
| CI=confidence intervals  Adjusted for age, sex, race, smoking, significant alcohol use, body mass index, total cholesterol, recreational physical activity, marital status, education attainment, and poverty income ratio.  13,529 participants were eligible for analysis | | | |

| Table S7. The associations of serum total bilirubin with lipid profile | | |
| --- | --- | --- |
| Serum total bilirubin | r | *P* value |
| Total cholesterol | 0.006 | 0.233 |
| High-density lipoprotein cholesterol | 0.015 | 0.003 |
| Low-density lipoprotein cholesterol | 0.008 | 0.282 |
| Triglyceride | -0.019 | 0.015 |
| 16,280 participants were eligible for analysis | | |

**A**

C-reactive protein

β=0.045

β=-0.009

β=-0.002

Serum glucose

Serum total bilirubin

**B**

C-reactive protein

β=0.033

β=-0.009

β=-0.009

Glycohemoglobin

Serum total bilirubin

Figure S1. The mediation model of c-reactive protein in the association of serum total bilirubin with glucose and glycohemoglobin. A: serum glucose; B: glycohemoglobin. In the mediation analysis, patients with self-reported use of insulin or other diabetes were excuded, finally, 14,449 paricipants with c-reactive protein data were used.
